# Bimodal inguinal sentinel lymph node imaging using a radiation-free fluorescent magnetic hybrid tracer in penile cancer patients

**DOI:** 10.3389/fonc.2025.1523038

**Published:** 2025-05-09

**Authors:** Bianca Michalik, Svenja Engels, Maximilian C. Otterbach, Martin H. Maurer, Friedhelm Wawroschek, Alexander Winter

**Affiliations:** ^1^ University Hospital for Urology, Klinikum Oldenburg, Department of Human Medicine, School of Medicine and Health Sciences, Carl von Ossietzky University Oldenburg, Oldenburg, Germany; ^2^ University Institute for Diagnostic and Interventional Radiology, Klinikum Oldenburg, Department of Human Medicine, School of Medicine and Health Sciences, Carl von Ossietzky University Oldenburg, Oldenburg, Germany

**Keywords:** fluorescence imaging, hybrid tracer, ICG, penile cancer, sentinel lymph nodes, SPION

## Abstract

Invasive lymph node (LN) staging is crucial for survival in penile cancer (PeCa) patients. To lower patient morbidity associated with radical inguinal lymphadenectomy, sentinel node biopsy (SNB) is recommended. Application of conventional radioactive/fluorescent tracers for sentinel node (SN) labelling is limited to centers with nuclear medicine or lacks pre-operative imaging. We introduce a radiation-free fluorescent magnetic hybrid tracer for bimodal inguinal SN imaging in PeCa patients. In three consecutive PeCa patients, the fluorescent magnetic hybrid tracer (50 µl indocyanine green, 5 mg/ml, in 1 ml superparamagnetic iron oxide nanoparticles) was peritumorally injected. SNs were visualized by magnetic resonance imaging (MRI). Intra-operatively, SNs were detected using a handheld magnetometer and a fluorescence camera. Concordance was determined between MRI and magnetometer-guided SNB and between magnetic and fluorescent SN labelling. MRI revealed 29 SNs (median 4.5, range 0–8 SNs/groin). Twenty-five LNs (median 4.5, range 0–9 LNs/groin) were resected, including 16 magnetically active and 17 fluorescent SNs (median 3, range 0–6 SNs/groin, either mode). MRI and magnetometer-guided SNB had 66% concordance, magnetic and fluorescence SN labelling 96%. The diagnostic accuracy of our approach has to be evaluated in larger patient cohorts. Our radiation-free SNB technique is feasible without the need for nuclear medicine, its associated additional effort and regulations.

## 1 Introduction

In penile cancer (PeCa) patients, early and correct inguinal lymph node (LN) staging is crucial for prognosis and therapy planning as it directly affects the patients’ survival (1). Invasive LN staging is the preferred option (2) as imaging techniques lack sensitivity for detecting inguinal metastases smaller than 10 mm (1). Still, up to 55% of PeCa patients are subjected to post-operative morbidity with the number of resected LNs as main predictor for complications (1). European guidelines (1) therefore highly recommend sentinel node biopsy (SNB) for inguinal LN staging. The concept behind SNB is that sentinel nodes (SN) represent the first lymphatic drainage stations of a tumor bearing organ and carry thus the highest risk of LN invasion (LNI) (1). Hence, by identification and targeted resection of SNs a high staging accuracy can be achieved while reducing the risk of post-surgical complications. In PeCa, SNB has developed to a safe and reliable LN staging technique, particularly when using an indocyanine green-^99m^Technetium (ICG-^99m^Tc) hybrid tracer for SN marking combined with pre-operative lymphoscintigraphy as well as single-photon emission computed tomography/CT (SPECT/CT) (3, 4). The addition of ICG to the conventional tracer has been found to be especially helpful due to intra-operative optical SN detection (3). This procedure carries, however, the disadvantage of radioactivity and its general application may therefore be limited to specialized centers with nuclear medicine infrastructure. Radiation-free inguinal SN marking can be achieved by superparamagnetic iron oxide nanoparticles (SPION) (5). Pre-operative imaging and surgical planning can be realized via magnetic resonance imaging (MRI) (6). A combination of ICG and SPIONs for inguinal SN imaging and surgery has already been introduced in animal models (7). After successful application in prostate cancer patients (8), we present first results of the use of a fluorescent magnetic hybrid tracer for bimodal inguinal SN imaging in PeCa patients.

## 2 Methods

To demonstrate clinical tracer applicability, we included three consecutive PeCa patients with ≥ pT1a G2 tumors who were scheduled for inguinal SNB according to the European guidelines (1) at our center in 2023. We peritumorally injected the ICG-SPION hybrid tracer consisting of 50 µl ICG solution (25 mg ICG powder (Verdye, Diagnostic Green, Aschheim-Dornach, Germany) dissolved in 5 ml sterile water) and 1 ml SPION (Magtrace, Endomag, Cambridge, UK) one to three days before surgery. All patients were informed orally and in writing about the procedure and possible associated risks and all patients gave written informed consent. Abdominal transversal T1-, T2-, and T2*-weighted MRI sequences (6) were recorded for pre-operative inguinal SN visualization at four to seven hours before surgery. A radiologist well-experienced in magnetic sentinel diagnostics using MRI evaluated the scans and counted SNs for each groin separately. During SNB, each groin was systematically searched for magnetic as well as fluorescence signal using a handheld magnetometer probe (Sentimag, Endomag, Cambridge, UK) and a near-infrared fluorescence imaging (FI) system (Quest SPECTRUM 3, Olympus, Hamburg, Germany), respectively. LNs closely adjacent to SNs or suspicious LNs identified by the surgeon were resected, too. All resected LNs were documented, re-measured ex vivo for magnetic activity as well as for fluorescence, and sent to conventional histopathology as individual samples.

## 3 Results

**Figure 1:**
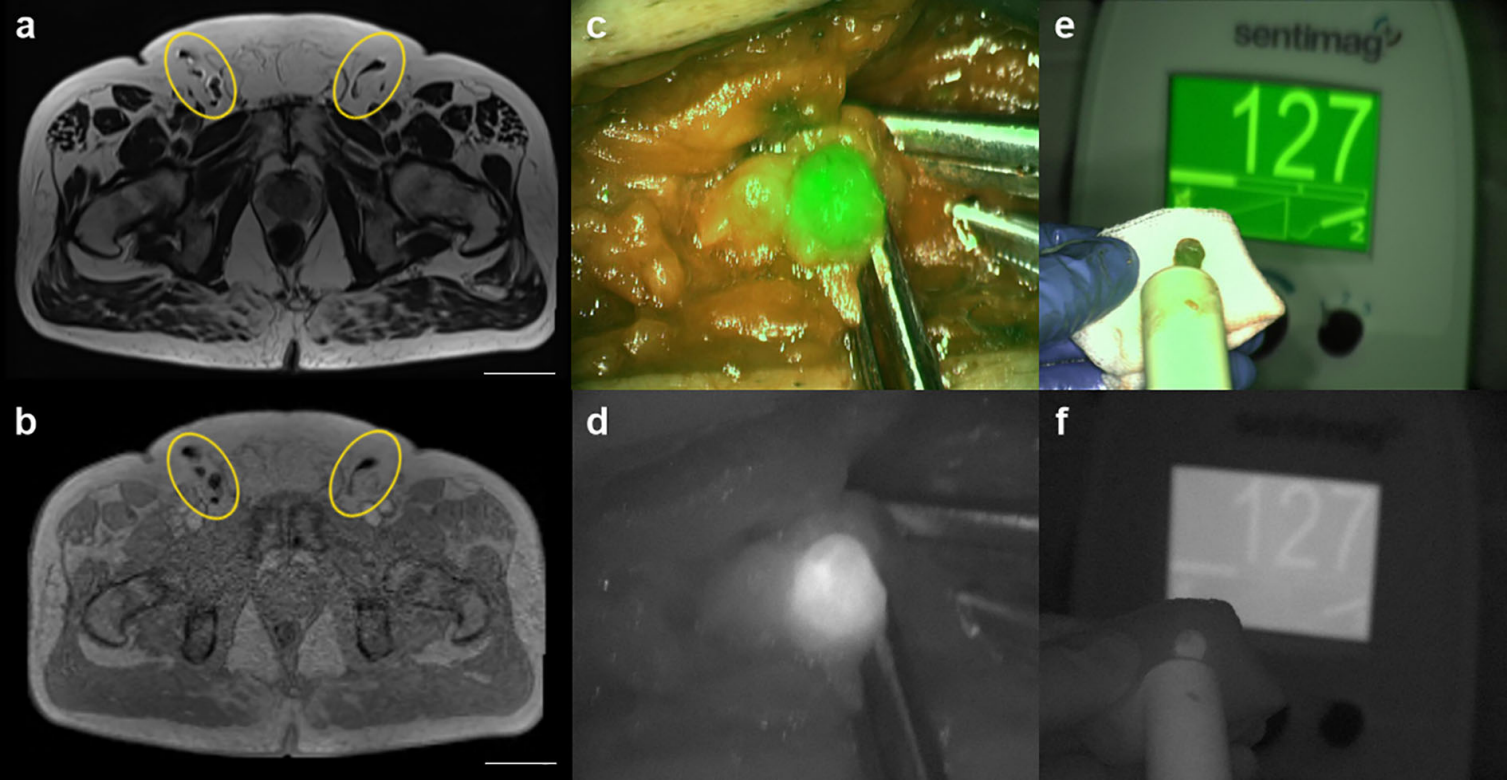
Pre- and intra-operative bimodal inguinal sentinel node (SN) imaging. **(a, b)** transversal abdominal T2- and T2*-weighted MRI scans (scale 50 mm) for pre-operative inguinal SN visualization (yellow circles). **(c, d)** intra-operative optical SN detection using a fluorescence imaging (FI) system; **(c)** overlay, **(d)** laser light. **(e, f)** resected fluorescent and magnetically active SN recorded by handheld magnetometer probe and FI; **(e)** overlay, **(f)** laser light.

Clinical patient characteristics as well as therapeutic data are summarized in **Table 1**. On pre-operative inguinal MRI scans (**Figures 1a, b**), 29 LNs (**Table 1**) with SPION uptake have been identified. Overall, 25 LNs have been resected during inguinal SNB of which 16 LNs were magnetically active (**Table 1**). Concordance between pre-operative inguinal MRI scans and magnetometer-guided SNB was 66%. FI revealed, in total, 17 fluorescent LNs (**Figures 1c, d**; **Table 1**). All but one fluorescent LNs were magnetically active as well (**Figures 1e, f**). The resulting concordance between magnetometer-guided and FI-guided SNB was 96%. An example of a resected fluorescent as well as magnetically active SN is shown in **Figure 1f**. All resected LNs were histopathologically negative.

**Table 1:** Clinical patient characteristics and therapeutic data.

Clinical characteristics	
Age at SNB (years)	74 (73–77)
BMI (kg/m²)	29 (24–35)
Primary tumor excision (days prior SNB)	29 (27–34)
cN status	0
Pathological characteristics	
Tumor location	glans penis
Infiltration depth (mm)	2.2 (1.1–2.5)
T-category (patients)	
1a	2
1b	1
Grade (patients)	
2	2
3	1
Lymphovascular invasion (patients)	0
Perineural invasion (patients)	0
Tracer administration (hours prior SNB)	30.20 (25.8–76.3)
Inguinal SNB	
SNs visualized on MRI (per groin)	4.5 (0–8)
LNs resected (per groin)	4.5 (0–9)
Magnetically active SNs (per groin)	3 (0–6)
Fluorescent SNs (per groin)	3 (0–6)

Data are numbers or medians (range). BMI, body mass index; cN, clinical nodal status; LN, lymph node; MRI, magnetic resonance imaging; SN, sentinel node; SNB, sentinel node biopsy.

None of the patients showed adverse reactions to tracer injection. No post-operative wound complications, such as seroma, infection or dehiscence, have been observed.

## 4 Discussion

These are the first results of radiation-free bimodal inguinal SN imaging using the new fluorescent and magnetic hybrid tracer in PeCa patients. The SPION component of the hybrid tracer enables pre-operative inguinal SN visualization and surgical planning via MRI as wells as intra-operative SN detection using a handheld magnetometer. The moderate concordance rates between MRI and intra-operative SN detection as observed in this pilot study could be partly explained by the high spatial resolution of MRI and its high sensitivity to very small concentrations of SPION. Pre-operative MRI might thus overestimate the actual SN number as we have reported previously for prostate cancer (8). To improve comparability between pre- and intra-operative SN detection and between future studies as well, we recommend to set up uniform definitions to distinguish magnetic SNs from other magnetic activity as they are used for the radioactive procedure (10).

The ICG component of our new hybrid tracer enables optical SN identification during surgery and thus, meticulous SN resection. Concordance between fluorescence and magnetic SN labelling was 96%. Due to its small molecule size, free ICG tends to label not only SNs but also higher echelon nodes (9). As observed in the ICG-^99m^Tc hybrid tracer, the non-covalent binding of ICG to SPION might thus avoid unnecessary LN resection (10). This is crucial especially in intermediate risk (pT1a G2) patients for whom the risk of LN metastasis as well as the risks associated with surgical staging have to be carefully balanced (1). Our study is limited by its small sample size. While the number of only three patients is adequate to address the clinical application of our new hybrid tracer in this rare tumor entity, our results regarding the diagnostic accuracy of the new technique are only preliminary. The diagnostic accuracy as well as safety of the fluorescent magnetic hybrid tracer have to be confirmed in multicentric studies including larger patient series. In order to acknowledge possible undetected false negative procedures, future studies assessing the diagnostic accuracy of our new technique should also implement an oncological follow-up schedule for at least two years from surgery. Nevertheless, our radiation-free approach for SNB can easily be transferred to centers without nuclear medicine infrastructure and might help to improve surgical inguinal LN staging while reducing post-surgical morbidity in PeCa patients.

## Data Availability

The datasets generated and analyzed during the current study are available from the corresponding author on reasonable request.
